# Identification of Novel Astroviruses in the Gastrointestinal Tract of Domestic Cats

**DOI:** 10.3390/v12111301

**Published:** 2020-11-12

**Authors:** Kate Van Brussel, Xiuwan Wang, Mang Shi, Maura Carrai, Jun Li, Vito Martella, Julia A. Beatty, Edward C. Holmes, Vanessa R. Barrs

**Affiliations:** 1 School of Veterinary Science, Faculty of Science, University of Sydney, Sydney, NSW 2006, Australia; kate.vanbrussel@sydney.edu.au (K.V.B.); julia.beatty@sydney.edu.au (J.A.B.); 2 Marie Bashir Institute for Infectious Diseases and Biosecurity, School of Life and Environmental Sciences and School of Medical Sciences, University of Sydney, Sydney, NSW 2006, Australia; mang.shi@sydney.edu.au (M.S.); edward.holmes@sydney.edu.au (E.C.H.); 3 Department of Infectious Diseases and Public Health, Jockey Club of Veterinary Medicine and Life Sciences, City University of Hong Kong, Kowloon Tong, Hong Kong, China; xiuwan.wang@my.cityu.edu.hk (X.W.); jun.li@cityu.edu.hk (J.L.); 4 School of Medicine, Sun Yat-sen University, Guangzhou 510275, China; 5 Department of Veterinary Clinical Sciences, Jockey Club College of Veterinary Medicine and Life Sciences, City University of Hong Kong, Kowloon Tong, Hong Kong, China; mcarrai@cityu.edu.hk; 6 School of Data Science, City University of Hong Kong, Hong Kong, China; 7 Department of Veterinary Medicine, University of Aldo Moro of Bari, 70010 Valenzano, Italy; vito.martella@uniba.it

**Keywords:** feline astrovirus, diarrhoea, mamastrovirus, capsid, domestic cats, evolution

## Abstract

Astroviruses, isolated from numerous avian and mammalian species including humans, are commonly associated with enteritis and encephalitis. Two astroviruses have previously been identified in cats, and while definitive evidence is lacking, an association with enteritis is suggested. Using metagenomic next-generation sequencing of viral nucleic acids from faecal samples, we identified two novel feline astroviruses termed Feline astrovirus 3 and 4. These viruses were isolated from healthy shelter-housed kittens (Feline astrovirus 3; 6448 bp) and from a kitten with diarrhoea that was co-infected with Feline parvovirus (Feline astrovirus 4, 6549 bp). Both novel astroviruses shared a genome arrangement of three open reading frames (ORFs) comparable to that of other astroviruses. Phylogenetic analysis of the concatenated ORFs, ORF1a, ORF1b and capsid protein revealed that both viruses were phylogenetically distinct from other feline astroviruses, although their precise evolutionary history could not be accurately determined due to a lack of resolution at key nodes. Large-scale molecular surveillance studies of healthy and diseased cats are needed to determine the pathogenicity of feline astroviruses as single virus infections or in co-infections with other enteric viruses.

## 1. Introduction

*Astroviridae* is a family of small (~6–7 kb), non-enveloped single-stranded RNA viruses that include two genera—*Avastrovirus* and *Mamastrovirus*. Species in the *Avastrovirus* genus infect avian hosts while those in the *Mamastrovirus* genus infect mammals including humans. As of 2019, the International Committee on Taxonomy of Viruses (ICTV) recognises 22 astrovirus species based on the capsid gene, three of which belong to *Avastrovirus* and the remainder to *Mamastrovirus* (https://talk.ictvonline.org/taxonomy/). Since then, an additional 33 and 7 candidate species have been discovered for the *Mamastrovirus* and *Avastrovirus* genera, respectively [1]. The astrovirus genome comprises three open reading frames (ORFs): ORF1a and ORF1b encode the non-structural proteins involved in viral replication, including the RNA-dependent RNA polymerase (RdRp), while ORF2 encodes the capsid precursor [2].

Human astroviruses are associated with acute gastroenteritis in all age groups, although they have also been reported in immunosuppressed children with encephalitis [3,4,5]. Astroviruses are also associated with enteritis in turkeys [6], hepatitis in ducks [7,8], nephritis in chickens [9], and encephalomyelitis in cattle [10] and mink [11,12]. Feline astrovirus (FAstV) was first discovered using electron microscopy in a kitten with diarrhoea in 1981 [13]. Since then, FAstVs have been detected in both clinically healthy cats and cats with diarrhoea in multiple countries using electron microscopy, viral culture and/or molecular methods [14,15,16,17,18,19,20]. Genome sequencing has revealed at least two groups of genetically distinct FAstVs. The species *Mamastrovirus 2* includes Feline astrovirus 2 and is related to classic human astroviruses (*Mamastrovirus 1*). In contrast, strain FAstV D1 clusters with fox, California sea lion, and mink astroviruses [14,16], suggesting that cats may harbour a variety of astrovirus strains that have not been studied in terms of their pathobiological properties.

Herein, we used metagenomic next-generation sequencing (mNGS) of viral nucleic acids isolated from faecal samples to identify two novel FAstVs, one detected in three clinically healthy kittens and another isolated from a kitten with diarrhoea that was co-infected with Feline parvovirus (FPV), a virus in the species *Carnivore protoparvovirus 1*.

## 2. Materials and Methods

### 2.1. Ethics

The collection of faecal samples from cats in this study was approved by the University of Sydney Animal Ethics Committee (AEC approval number N00/7-2013/3/6029).

### 2.2. Sample Collection

Faeces were collected from an unvaccinated 8-week-old male shelter-housed kitten with diarrhoea (cat #159) during an outbreak of feline panleukopenia (FPL) at a shelter in Sydney, New South Wales, Australia [21]. The kitten tested positive for FPV on a faecal antigen test and FPV infection was confirmed on Sanger sequencing of the FPV VP2 gene [21]. Faecal samples were also collected from three clinically healthy domestic shorthair (DSH) kittens aged 3 to 5 months (cat #AWL4, #AWL6 and #AWL8) from a second shelter in Sydney, New South Wales, Australia. All faecal samples were stored at −80 °C until processing.

### 2.3. Viral Nucleic Acid and Total RNA Isolation and Sequencing

Viral nucleic acids were isolated from the faecal samples of all cats using a previously published protocol for viral particle enrichment [22] with minor modifications [23]. The QIAamp Viral RNA Mini Kit (Qiagen, Hilden, Germany) was used to extract viral nucleic acids that were treated with DNase (Invitrogen, Thermo Fisher Scientific) to remove viral genomic DNA. The resulting viral genomic RNA was randomly amplified using the Whole Transcriptome Amplification Kit (WTA2) (Sigma-Aldrich, St. Louis, MO, USA) and 22 PCR cycles [22,23] to convert RNA to cDNA then purified using the GenElute PCR Clean-up Kit (Sigma-Aldrich, St. Louis, MO, USA). cDNA quantity was evaluated using the Qubit 2.0 fluorometer. The Nextera XT DNA Library Preparation Kit (Illumina, San Diego, CA, USA) was used to produce the sequencing libraries that were then sequenced on the NovaSeq6000 (Illumina, San Diego, CA, USA) platform (150 bp paired end) at the Australian Genome Research Facility (AGRF) (Melbourne, Australia). Insufficient viral genomic RNA was extracted from the faecal sample from cat #159 using the viral particle enrichment protocol such that a cDNA library could not be synthesized for sequencing. Therefore, in this case, total RNA was isolated from this sample using the RNeasy Plus Mini Kit (Qiagen, Hilden, Germany) following manufacturer instructions. Prior to total RNA isolation, the faecal sample was suspended in 600 µL lysis buffer and homogenized at 5 ms^−1^ for 1.5 min using the Omni Bead Ruptor (Omni international, Kennesaw, GA, USA) [23]. Total RNA quality and quantity were assessed using a Bioanalyzer 2100 (Agilent, Santa Clara, CA, USA). The Zymo-Seq RiboFree Total RNA Library Kit (Zymo Research, Irvine, CA, USA) was used for rRNA depletion and library preparation and sequencing was performed on the NovaSeq6000 (Illumina, San Diego, CA, USA) platform (150 bp paired end) at the AGRF (Melbourne, Australia).

### 2.4. Genome Assembly and Read Mapping

Raw reads were mapped to the cat genome (*Felis catus* 9.0 assembly, GenBank Assembly ID GCA_000181335.4) with BWA version 0.7.17 [24] and reads with over 95% mapping coverage were removed. The ribosomal RNA (rRNA) reads were filtered with SortMeRNA [25]. Raw reads were further processed for quality control using the following procedures [26,27]: (available at https://github.com/TingtZHENG/metagenomics/blob/master/scripts/fqc.pl): (i) removal of Illumina primers/adaptors/linker sequences; (ii) removal of paired ends reads with 25 bp consecutively exact match from both ends to avoid PCR duplicates; and (iii) removal of terminal regions with continuous Phred based quality <20. After pre-processing of raw data, IDBA-UD version 1.1.2 [28] was used for de novo assembly of the cDNA libraries from cats #AWL4, #AWL6 and #AWL8. To confirm the IDBA-UD assembly accuracy de novo, assembly of the cDNA libraries was also performed using Trinity version 2.8.5 [29]. For cat #159, Trinity [29] was used for the de novo assembly of the total RNA sequencing reads. Diamond version 0.9.32 was used to compare the resulting contigs to the NCBI non-redundant protein database. Viral read counts for cDNA libraries after quality control filtering and IDBA-UD assembly for cats #AWL4, #AWL6, and #AWL8 were confirmed using BWA [24]. BWA was also used to determine the viral read count for the meta-transcriptomic library after quality control filtering and Trinity assembly for cat #159 [24]. Astrovirus genomes were annotated and ORFs were predicted using Geneious version 2019.2.1. Transmembrane domains were predicted using TMHMM version 2.0 (http://www.cbs.dtu.dk/services/TMHMM/) and the NCBI Conserved Domain Database (CDD) was used to predict astrovirus genome motifs and domains. All astrovirus genomes described here have been deposited on GenBank under the accession numbers MW037839-41.

### 2.5. Evolutionary Analysis

Phylogenetic analysis of *Mamastrovirus* genomes was performed on the individual amino acid sequence alignments of the ORF1a, ORF1b, and capsid genes, as well as on a concatenated alignment of all three ORFs. For each gene, between 39 and 48 reference sequences of mamastroviruses were included to represent the background diversity of these viruses in mammals (reference sequences were downloaded from GenBank, excluding accession numbers MN977118-9). Sequence alignments were performed in Geneious using MAFFT version 7 and the E-INS-i algorithm [30], and GBlocks was used to remove ambiguously aligned regions and gaps [31,32]. This resulted in final amino acid alignments of 816, 230, 373, and 215 residues for the concatenated ORFs, ORF1a, ORF1b, and capsid genes, respectively. The PhyML program was used to estimate the maximum likelihood phylogenetic trees assuming the Le and Gascuel 2008 (LG) and gamma distribution (Γ) model of amino acid substitution, subtree pruning and regrafting (SPR) branch swapping, and bootstrap re-sampling (1000 replicates) to assess nodal support [33,34,35].

A nucleotide sequence alignment containing 39 *Mamastrovirus* reference sequences and the FAstVs identified here was created using MAFFT. The RDP, GENECOV, and Bootscan programs within the RDP4 package [36] were then used to scan this alignment for potential recombination events involving FAstV3 and FAstV4. The genomic locations of any putative recombination breakpoints were then determined using Simplot, with new (amino acid) phylogenetic trees then inferred at either side of these recombination breakpoints using the phylogenetic procedure described above.

## 3. Results

### 3.1. Genome Features of Novel FAstVs

**Figure 1:**
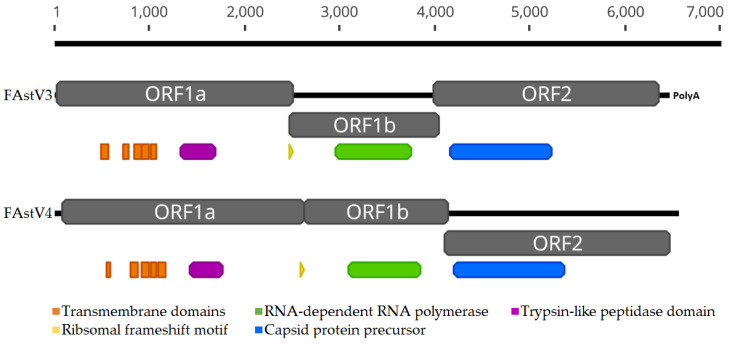
An overview of the genome structures of FAstV3 and FAstV4. ORFs are represented in grey, transmembrane domains in orange, trypsin-like peptidase domain in purple, RdRp in green, and capsid protein precursor in blue. The yellow triangle represents the ribosomal frameshift motif. ORF1a, ORF1b, and ORF2 are 832, 519 and 786 amino acids for FAstV3 and 848, 506 and 789 amino acids for FAstV4, respectively.

Using mNGS, we identified two novel FAstVs of lengths 6448 bp (excluding the 10 bp polyA tail) and 6549 bp (in which no polyA tail was observed). The first FAstV (6448 bp), tentatively named Feline astrovirus 3 (FAstV3), was detected in three healthy cats, including whole genomes in two (#AWL4 and #AWL6) and contigs of lengths 493 bp (ORF1a) and 517 bp (ORF2) and 100% identity in the third (#AWL8). Both assemblers showed comparable results, although because Trinity added a small section of sequence to the 3′ end of FAstV3 after the polyA tail we choose the IDBA-UD assembly in this case. The second novel FAstV (6549 bp), tentatively named Feline astrovirus 4 (FAstV4), was sequenced from the sick cat (#159). The three ORFs were predicted for both FAstVs, and a polyA tail was only observed in FAstV3 (Figure 1). The GC content for FAstV3 was 50% and 45.3% for FAstV4. The ribosomal frameshift AAAAAAC motif was present in the ORF1a and ORF1b overlap in both genomes (Figure 1). TMHMM was used to predict five transmembrane domains in FAstV3 at amino acid positions 161–183, 237–256, 276–298, 302–324 and 331–353, and five in FAstV4 at positions 156–173, 243–265, 285–307, 317–335 and 342–364 in ORF1a (Figure 1). The trypsin-like peptidase domain in ORF1a, RdRp in ORF1b, and the capsid protein precursor in ORF2 were identified (Figure 1).

### 3.2. Abundance, Sequence Comparison and Other Viruses

The assembled FAstV3 and FAstV4 genomes were used to map sequence reads from the four filtered sequencing libraries to determine read abundance (Table 1). BlastX revealed 61% amino acid sequence similarity to *Mamastrovirus 10* (GenBank accession number NC_004579) in the capsid gene for FAstV3 and 68% amino acid similarity to Canine astrovirus (GenBank accession number KX599354) in the capsid gene for FAstV4. FAstV3 and FAstV4 displayed 67% amino acid similarity to each other in the capsid gene. A complete *Mamastrovirus 2* genome (6796 bp excluding polyA tail, accession number MW037841) was detected in two cDNA libraries from cats #AWL4 and #AWL6 (Table 1) and shared 92% nucleotide similarity to other *Mamastrovirus 2* sequences and 97% (ORF1a and ORF1ab) and 94% (capsid gene) amino acid similarity to *Mamastrovirus 2* and Feline astrovirus 2, respectively.

Feline coronavirus reads were detected in all four filtered sequencing libraries, with one complete genome observed in the total RNA library from cat #159 (29432 bp) and contigs ranging in length from 6167 bp to 457 bp in library #AWL8. Additionally, a Feline picornavirus genome (7466 bp) was detected in the #AWL8 library.

### 3.3. Phylogenetic and Recombination Analysis

**Figure 2:**
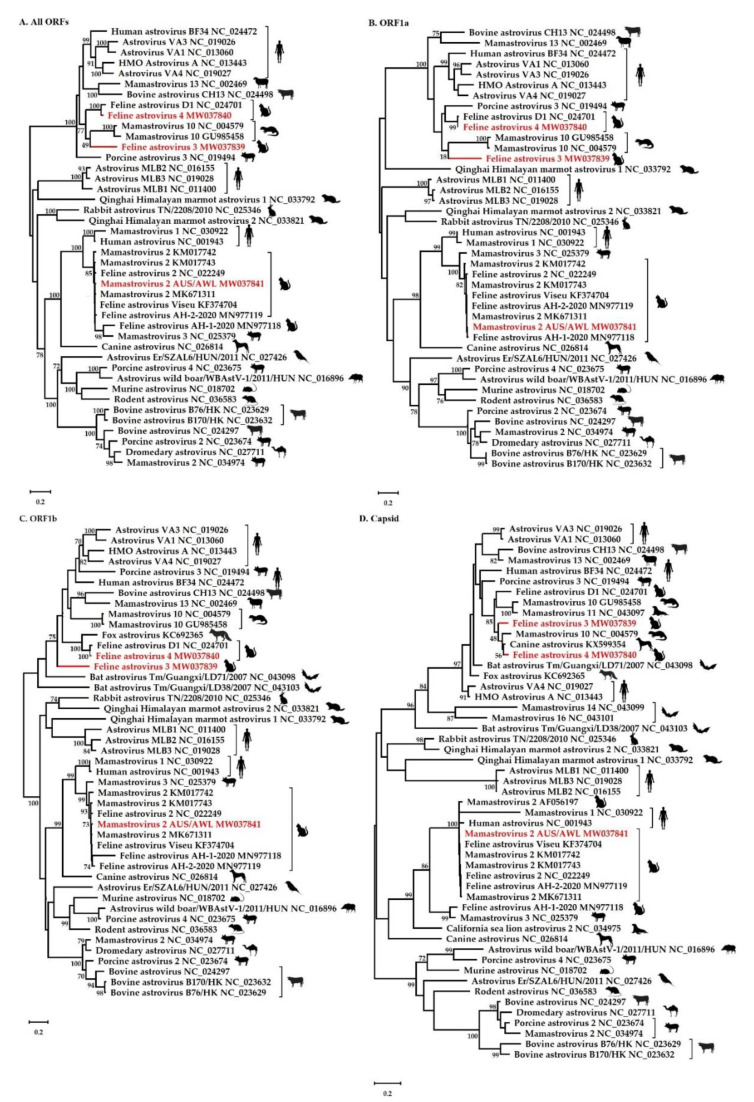
The phylogenetic relationships of the FAstVs described in this study to other Mamastrovirus species based on (**A**) concatenated ORFs, (**B**) ORF1a, (**C**) ORF1b, and (**D**) capsid gene sequences. All four trees are rooted at the midpoint, and bootstrap values are displayed next to the branches. GenBank accession numbers are listed for reference sequences after the organism name. The novel FAstVs and *Mamastrovirus 2* in this study are marked in red. Accession numbers NC_043099, NC_0431101, NC_034975, NC_043097, KX599354, and AF056197 are missing from the concatenated ORFs, ORF1a, and ORF1b (A, B and C) trees, and accession numbers KC692365, NC_043098, and NC_043103 are missing from the concatenated ORFs and ORF1a trees due to incomplete or unavailable gene sequences on GenBank.

Phylogenetic analysis of the concatenated sequences of all ORFs (Figure 2A) shows that both FAstV3 and FAstV4 cluster in a broad group of mamastroviruses, including FAstV D1, as well as astroviruses from humans, mink, and bovines. Although FAstV4 was clearly related to FAstV D1 with 100% bootstrap support in the concatenated ORFs, ORF1a, and ORF1b trees (and relatively close to FAstV D1 in the capsid gene phylogeny; see below), the precise phylogenetic position of FAstV3 was less certain because of a lack of strong (i.e., >70%) bootstrap support and topological differences between each gene tree. In particular, in the ORF1b phylogeny (Figure 2C), FAstV3, fell in a divergent and ambiguous phylogenetic position, and was also relatively divergent in the ORF1a tree (Figure 2B). Also of note was that in the phylogenetic analysis based on the capsid gene (Figure 2D), both FAstV3 and FAstV4 clustered with Canine astrovirus, *Mamastrovirus 10* (from mink), *Mamastrovirus 11* (from California sea lion) and FAstV D1, although again without strong bootstrap support.

Because there was some evidence for topological movement in the trees, compatible with the occurrence of recombination, we performed a more detailed analysis of recombination using RDP4 [36]. While this again revealed that FAstV3 and FAstV4 changed phylogenetic positions across the genome, phylogenetic trees estimated for sequence regions on either side of the putative recombination breakpoints were not significantly different (as measured by levels of bootstrap support >70%). Hence, there is no conclusive evidence for recombination in these data and any change in tree topologies may simply reflect a lack of phylogenetic resolution. Irrespective of this lack of phylogenetic resolution, FAstV3 and FAstV4 are clearly distinct from all other feline astroviruses.

## 4. Discussion

We used mNGS to detect two novel FAstVs in the gastrointestinal tract of kittens. The pathogenic potential of astroviruses in cats is not yet clearly understood. In humans, astroviruses have been shown to cause gastroenteritis and are responsible for up to 10% of non-bacterial gastroenteritis cases [3]. Similar to three previous reports, we identified one of the astroviruses (FAstV4) in a sick cat co-infected with FPV [17,20,37]. Since FPV causes severe gastroenteritis in cats, causality between FAstV infection and diarrhoea is difficult to ascertain. Previous evidence in support of a pathogenic role for FAstVs includes the induction of diarrhoea after experimental inoculation of SPF cats, as well as results of some molecular surveillance studies [37,38,39]. While one study found no significant difference in the prevalence of FAstV infection in cats with and without diarrhoea [40], another detected FAstVs in 38/105 (36%) cats with diarrhoea and in only 8/92 (8.7%) cats without diarrhoea [37]. In the latter study, co-infections of FAstV with FPV and/or Feline Bocavirus (FBoV) were present in 35/105 cats with diarrhoea (33%), whereas no FAstV co-infections were detected in cats without diarrhoea.

Co-infections of some enteric viruses in humans can increase the severity of clinical signs of acute gastroenteritis caused by single virus infections [41]. Whether FAstV and FPV or other enteric virus co-infections could contribute to more severe clinical disease than single virus infections warrants further investigation using large-scale NGS molecular surveillance studies of cats with and without enteric disease. All of the cats we investigated were also co-infected with feline coronavirus (FCoV), an enteric alpha-coronavirus. FCoV is ubiquitous in most multicat environments and was detected in all of 37 catteries tested in Germany recently [42]. FCoV most commonly exists as an avirulent pathotype that causes subclinical infection or occasionally mild enteritis but can mutate to acquire macrophage-tropism and cause the systemic inflammatory disease feline infectious peritonitis.

Phylogenetic analysis of the concatenated ORFs, ORF1a, ORF1b and capsid gene sequences revealed that despite some topological uncertainty, both FAstV3 and FAstV4 are clearly distinct from the established species *Mamastrovirus 2* and FAstV2. In some of the gene trees, we observed a broad grouping of FAstV3, FAstV4, a mink astrovirus associated with diarrhoea in farmed minks (Genbank accession NC_004579) and another associated with shaking mink syndrome (GenBank accession GU985458), a FAstV from healthy shelter-housed cats (GenBank accession NC_024701), and in the case of the capsid tree a canine astrovirus detected in the faeces of a shelter-housed dog with diarrhoea (GenBank accession KX599354) and a California sea lion astrovirus (GenBank accession NC_043097). However, while FAstV4 often groups with FAstV D1 (the only exception being the capsid gene tree), the exact phylogenetic position of FAstV3 is currently unresolved. It is possible that phylogenetic uncertainty in part reflects the action of recombination that has previously been documented in mammalian astroviruses [43,44,45,46], although we found no firm evidence for this process here.

The genetic diversity of astroviruses poses challenges for their diagnosis. For instance, there are at least five distinct Mamastrovirus species (1, 6, 8, 9 and the tentative species 20) in the human host and different assays are required for their molecular diagnosis, although commercial assays are available only for classical human astroviruses (*Mamastrovirus 1*) [3,47]. In cats, astroviruses are not included routinely in the diagnostic algorithms of infectious diseases, thus hindering the collection of useful information in terms of epidemiological and clinical data.

In sum, these findings support that FAstVs are part of the gastrointestinal virome of domestic cats and demonstrate the extensive genetic diversity of FAstVs. Whether there are biological differences among the various FAstV strains should be considered, as astroviruses exhibit considerable biological plasticity. Accordingly, it is important to determine whether some FAstVs may act as either primary pathogens or opportunists.

## Figures and Tables

**Table 1:** Summary of library and virus read counts, transcripts per million counts for each virus, and the assembler used.

Virus	Accession Number	Assembler	Library Virus Was Identified	Library Read Count after Processing (Paired End)	Virus/Contig Read Count	Transcripts per Million
Feline astrovirus 3	MW037839	IDBA-UD	AWL4	7,030,294	62,663	1623
			AWL6	10,116,484	240,905	3213
			AWL8	10,578,534	222	32
					279	42
Feline astrovirus 4	MW037840	Trinity	159	77,205,956	32,003	16
Mamastrovirus 2 AUS/AWL	MW037841	IDBA-UD	AWL4	7,030,294	1636	70
					1897	114
			AWL6	10,116,484	4098	53
